# Effects of dietary supplementation with a microalga (*Schizochytrium* sp.) on the hemato-immunological, and intestinal histological parameters and gut microbiota of Nile tilapia in net cages

**DOI:** 10.1371/journal.pone.0226977

**Published:** 2020-01-02

**Authors:** Felipe Pinheiro de Souza, Ed Christian Suzuki de Lima, Angela Maria Urrea-Rojas, Suelen Aparecida Suphoronski, César Toshio Facimoto, Jailton da Silva Bezerra Júnior, Thalita Evani Silva de Oliveira, Ulisses de Pádua Pereira, Giovana Wingeter Di Santis, Carlos Antonio Lopes de Oliveira, Nelson Mauricio Lopera-Barrero

**Affiliations:** 1 Department of Animal Science, State University of Londrina, Londrina, Parana, Brazil; 2 Department of Preventive Veterinary Medicine, State University of Londrina, Londrina, Parana, Brazil; 3 Department of Animal Science, State University of Maringa, Maringa, Parana, Brazil; Universidad Miguel Hernández de Elche, SPAIN

## Abstract

Nutritional improvements in intensive aquaculture production systems is necessary for the reduction of stress, maximum utilization of nutritional components, and expression of the genetic potential of fish. The objective of this study was to evaluate the hemato-immunological, and histological parameters and gut microbiota of Nile tilapia fed with the microalga *Schizochytrium* sp. Males of Nile tilapia were distributed among eight net cages (6 m^3^), and fed for 105 days with two diets: control (CON), without *Schizochytrium* sp., and supplemented (SUP), with 1.2% *Schizochytrium* sp. in the diet. The final weight, mortality, hematocrit, total erythrocyte count (RBC), hemoglobin, hematimetric indices, white blood cell count (WBC), total protein, and serum lysozyme were measured. Alterations in intestinal morphology were evaluated. The gut microbiota was evaluated with next-generation sequencing. No significant differences (p>0.05) were found in the final weight and mortality between diets. Regarding the hematological parameters, a difference (p<0.05) was detected only in RBC, with there being lower values in the SUP, although this group also showed a tendency toward having an increased mean corpuscular hemoglobin level. There were no differences (p>0.05) in total protein and serum lysozyme concentrations or in WBCs between diets, except for lymphocytes, which presented lower values (p<0.05) in the SUP, suggesting immunomodulation by the polyunsaturated fatty acids present in the microalga. There was no difference (p>0.05) in the intestinal morphology between diets. Metagenomic data indicated greater richness (represented by the Chao index) and a higher abundance of the bacterial phylum Firmicutes in the gut microbiota of the tilapia fed with the SUP diet, demonstrating that the digestion and use of the components of the microalga could influence the microbial community. The results indicated that the microalga had modulatory effects on blood cells and the intestinal microbiota, without affecting the structure and integrity of the intestinal villi.

## Introduction

In recent decades, world aquaculture has undergone a marked expansion due to the increased production of some species, such as Nile tilapia (*Oreochromis niloticus*), which stands out as one of the most produced fish in the world [1]. In the year 2016, world production of Nile tilapia reached 4.2 million tons, representing 8% of the total fish produced in the world, with it being the fish produced at the highest levels in several countries [1]. In Brazil, the production of this species represented approximately half of all aquaculture production in 2017, which was approximately 559 thousand tons [2]. However, due to the rapid expansion and intensification of Nile tilapia production, the high densities adopted in farming systems have become a challenge, since they are a stress factor for the fish that can cause economic losses [3–5].

Given the above, Nile tilapia farming in intensive systems, such as net cages, has increased significantly. This growth has mainly been due to the excellent productivity per unit of space and ease of assembly of the cage structure, as well as the control of the stock and harvest it allows [6,7]. Because of the high density adopted in these tanks, the use of food that improves fish health and immunity is essential to minimize the effects of stress and allow the genetic potential of the fish to be fully expressed. In addition, there is a global demand for the use of low-cost natural compounds in animal feed that have physiological benefits and pose no risk to the environment [8].

Algae, being the bases of many natural aquatic food chains, represent an important food resource that contains several compounds with functional activities that can aid in animal and human nutrition [9]. Among the algae used for animal feed, the microalga *Schizochytrium* sp. stands out. This alga is particularly well-known for having a high content of polyunsaturated fatty acids (PUFAs), in particular docohesaexanoic acid (DHA) [10], which is known to have diverse human and animal health benefits. Studies have shown that the inclusion of *Schizochytrium* sp. in the diet can increase the concentration of fatty acids in Nile tilapia [11] and channel catfish (*Ictalurus punctatus*) [12], modulate the gut microbiota of rainbow trout (*Oncorhynchus mykiss*) [13], and increase the final biomass of Nile tilapia [14]. However, no studies have evaluated the effects of this microalga on fish farmed in intensive systems like net tanks.

Recently, technologies such as next-generation sequencing (NGS) have allowed there to be a rapid expansion in research related to fish nutrition. It is known that the microbiota of the digestive tract play important roles in the production of vitamins, nutrient distribution, regulation of innate immunity, and maintenance of intestinal tissue integrity [15,16], which can be modulated by different diets and farming conditions [17]. However, no study to date has been conducted in an integrated manner to evaluate the influence of dietary microalgae on intestinal histology and microbiota, in addition to on the general health and immunity of Nile tilapia. For these purposes, information on hematological and immunological parameters are useful to indicate the conditions of cellular defense and homeostasis during stressful periods, and previous studies have already shown that they can be modulated by the inclusion in the diet of algae, such as *Laminaria digitata* and *Ascophyllum nodosum* [18], *Ulva clathrata* [8] and *Gracilaria verrucosa*, which has also been shown to to be capable to increase the resistance of tilapia to pathogens [19]. In addition, PUFAs have been shown to influence immune response through modulation of leukocyte cells [20,21], although immunological studies evaluating the effects of providing PUFA-rich sources on fish feed are still scarce. Therefore, hematological and immunological assays on the effects of microalgae *Schizochytrium* sp may provide important information on a possible modulation of the immune response.

Therefore, the objective of the present study was to analyze the influence of the addition of the microalga *Schizochytrium* sp. to the diet on the hemato-immunological, and histological parameters and intestinal microbiota (assessed via NGS) of Nile tilapia cultivated in net cages.

## Materials and methods

The methodologies employed during this experiment were approved by the Ethics Committee on the Use of Animals of the State University of Londrina (CEUAUEL, n°18363.2017.07).

### Experimental design

This study was conducted in a fish farm from the Fish Experimental Station of the Maringá State University (UEM/Codapar) located in the Corvo River, near the city of Diamante do Norte and at the Rosana Hydroeletric plant (22°39'25.20" S and 52°46'52.78" W) in the state of Paraná, Brazil. Were used 800 Nile tilapia juveniles of the GIFT lineage that had been sexually reversed into males, with initial weights of approximately 50 g. The fish were distributed among eight net cages (6 m^3^) arranged in a completely randomized design, consisting of two treatments with four replicates each, with 100 fish per replicate for a total of 400 fish per treatment. The net cages were arranged in a single line while intercalating the treatments to avoid any effects of the position of the cage in the river.

To meet the study objectives and experimental conditions proposed, two treatments were used: one with a control diet (CON) (commercial feed without supplementation), and one using a diet supplemented with 1.2% microalgal (*Schizochytrium* sp.) meal (SUP) (guarantee levels per kilogram: Ether extract 500 g, DHA 140 g. Source: manufacturer–ALL-G RICH^™^, Alltech, Lexington, KY, USA). The microalgal meal was added to the extruded commercial feed, with soybean oil being used as the vehicle (1.6% of the diet in total). The control diet received only soybean oil in the same amount. The feeds were each mixed for five minutes in a concrete mixer (M-120 Maqtron). The procedures described by [22] were used for the calculation of the dry matter, crude protein, mineral matter, and crude energy (Parr Instrument Co. AC6200) in the ether extract of each feed (Table 1). Lipid extraction from the feed was performed by the Bligh-Dyer method [23]. The methyl esters of fatty acids were quantified according to the method of [24], in which the methyl ester of tricosanoic acid (Sigma, USA) was used as a standard. The theoretical values of the correction factor of the flame ionization detector (FID) were used to obtain the concentrations of fatty acids [25] (Table 1).

**Table 1:** Compositions of the control (CON) and alga-supplemented (SUP) diets provided to Nile tilapia.

**Centesimal composition**	**CON**	**SUP**
**Gross energy (kcal/kg)**	4.28	4.37
**Dry matter (%)**	92.33	92.46
**Crude protein (%)**	34.53	33.22
**Ether extract (%)**	5.41	6.10
**Mineral matter (%)**	11.51	11.52
**Lipid composition (mg/100 g of diet sample)**	**CON**	**SUP**
**PUFA**	1574.28	1970.87
**MUFA**	1352.37	1330.44
**SFA**	1113.69	1558.03
**FAT (%)**	4.44	5.34
**n-6/n-3**	250.08	12.95
**n-6**	1568.01	1829.57
**n-3**	6.27	141.30
**DHA**	Nd	131.81

PUFA = total polyunsaturated fatty acid; MUFA = total monounsaturated fatty acid; SFA = total saturated fatty acid; FAT = total fatty acid content; n-6 = total n-6 fatty acid; n-3 = total n-3 fatty acid; DHA = docosahexaenoic acid; nd = not detected. Composition: Soybean meal, ground whole corn, wheat meal, meat and bone meal, fish meal, degummed soybean oil. Vitamin and mineral mix (guarantee levels per kilogram): Calcium 15 g, Phosphorus 4000 mg, Sodium 2000 mg, Copper 15 mg, Manganese 40 mg, Zinc 60 mg, Cobalt 0.75 mg, Iodine 0.75 mg, Selenium 0.3 mg, Vitamin A 7000 UI, Vitamin D3 2000 UI, Vitamin E 90 UI, Vitamin K3 12 mg, Vitamin B1 20 mg, Vitamin B2 20 mg, Vitamin B6 20 mg, Vitamin B12 mcg, Niacin 100 mg, Calcium Pantothenate 50 mg, Folic Acid 5mg, Biotin 0.15, Vitamin C 300 mg, Choline Chloride 1350 mg, Inositol 30 mg. Source: manufacturer–Poli Nutri (Osasco, SP, Brazil).

An acclimatization period of 15 days was carried out, and then followed by the experimental period, which had a duration of 105 days (15 weeks). Monthly biometric analyses were performed to control the amount of feed provided. Fish were fed four times a day in the initial growth phase (5% of biomass, fish of 50-100g), two times a day in the growth phase (4% of biomass, fish of 100-500g), and two times a day in the final phase (2% of biomass, fish of 500g until end of the trial). Mortality was measured throughout the entire experimental period. The water temperature was evaluated daily in the morning and afternoon.

The final weight (g), standard length (cm) (from the anterior end of the head to the beginning of the caudal fin insertion), and total length (cm) (from the anterior end of the head to the end of the caudal fin) [26] of the fish was measured on the day of collection (105^th^ day). Condition factor (CF;%) was determined according [27], with the formula CF (%) = [weight of fish (g)/ (length of fish (cm))^3^] × 100.

### Hematological profile and total protein and serum lysozyme content

Samples of blood (1 mL) were collected from the caudal vein of the fish in plastic microtubes containing dipotassium ethylenediaminetetraacetic acid (K2EDTA, Hemstab, Lagoa Santa, MG, Brazil) as an anticoagulant for the preservation of the samples destined for use in hematological tests. Fourteen fish were used per diet group (CON and SUP), and blood was collected after the fish had been anesthetized with clove oil (0.1 ml L^-1^) (Eugenol, Ibipora, PR, Brazil). The determination of the total hemoglobin (Hb) concentration in the blood was performed by the hemoglobin cyanide method using the commercial Labtest^®^ kit (Lagoa Santa, MG, Brazil). Erythrocyte counts (RBC x 10^6^ μL^-1^) were made with a Neubauer camera after dilution (1:200) in Dacie’s solution [28]. For the calculation of the globular volume (VG in %) by the microhematocrit technique and the determination of the hematimetric indices MCV (mean corpuscular volume—fL), MCHC (mean corpuscular hemoglobin concentration—g dL^-1^), and MCH (mean corpuscular hemoglobin—pg), the methodologies described by [29] were followed. Leukocyte differential counts were performed using the indirect method with May-Grünwald-Giemsa-Wright staining [29]. Briefly, in each blood sample, 2000 cells were counted, including erythrocytes, thrombocytes, and leukocytes. The number of leukocytes (×10^3^ uL^-1^) (lymphocytes, neutrophils, and monocytes) and thrombocytes (×10^3^ uL^-1^) were calculated using a rule of three, while considering the number of cells counted in the neubauer camera.

To measure total serum proteins (g dL^-1^) and serum lysozyme (μg mL^-1^) concentrations in blood samples, 1 mL of blood (without anticoagulant) was centrifuged for 10 min at 2500× *g* for serum separation. Total serum protein was measured by the colorimetric biuret method (Analisa^®^, Belo Horizonte, MG, Brazil). Serum lysozyme activity was determined using a methodology adapted from that of [30]. Briefly, the initial and final absorbances were measured by spectrophotometry while determining the serum lysozyme activity by the lysis of the Gram-positive bacterium *Micrococcus lysodeikticus* (Sigma-Aldrich Chemical Co.). The reduction in the absorbances of the samples was converted into an estimate of the lysozyme concentration (μg mL^-1^). Both analyses were performed using a digital spectrophotometer (Coleman 33D).

### Intestinal histology

For this analysis, a medullary section was performed on the animals previously anesthetized for blood collection. Samples were collected from the distal intestine of 16 animals per treatment. The collected material was fixed in 10% buffered formaldehyde for 24 h, and then inserted into 70% ethanol. The samples were next submitted to serial dehydration in ethanol, sectioned into 4–5 μm sections, stained with Alcian blue (pH = 2.7) and hematoxylin and eosin (H&E), and covered with coverslips.

A semi-quantitative score was developed based on the proposed score of [31] for Atlantic salmon (*Salmo salar* L.) (Table 2). Briefly, five parameters were independently classified: 1) flattening of mucosal folds (MF); 2) width of the lamina propria (LP); 3) presence of supranuclear vacuoles (SNV); 4) abundance of goblet cells (GC); and 5) degree of infiltration of eosinophilic granulocytes (EG) into the lamina propria (LP) and subepithelial mucosa (SM). Each of these parameters was scored on a scale from 1 to 3, with lower score value representing a more normal condition and greater integrity of the villi and intestinal cells. The slides were visualized under an Opticam 0500R microscope coupled to a camera. The images were captured using OPTHD software (version x64 3.7.8).

**Table 2:** Description of the semi-quantitative score used to evaluate different histological parameters in the Nile tilapia intestine.

Parameter	Condition	Score
**Flattening of mucosal folds (MF)**	Basal length	1
	Diffused shrinkage and onset of tissue disruption	2
	Diffused or total tissue disruption	3
**Width of lamina propria (LP)**	Normal size, LP with a thin and delicate core of cells	1
	Increased size of LP	2
	Largest LP	3
**Presence of supranuclear vacuoles (SNV)**	Basal SNV size, normally aligned	1
	Diffuse size reduction, non-aligned	2
	Onset of extinction or no SNV	3
**Abundance of goblet cells (GC)**	Scattered cells, in normal amount	1
	Diffused numbers, widely spread out, GC increased	2
	Highly abundant, densely grouped cells	3
**Degree of infiltration of eosinophilic granulocytes (EG) into LP and SM**	Few in subepithelial mucosa (SM), basal, some migration into LP	1
	Diffuse number in SM, increased migration into LP	2
	Dense EG in LP and SM	3

Adapted from the score used by Uran et al. (2008) for Atlantic salmon (*Salmo salar* L.)

All statistical analyses were performed using the software R version 3.3.3 [32]. Data were presented as means ± standard error. The zootechnical indexes of the animals, results of hematological analyses, lysozyme concentration, total serum protein, and histological parameters were compared between diet groups using Student’s *t*-test, with a significance level of 5%. The data were submitted to Levene’s test to verify the homogeneity of their variances, and the Shapiro-Wilk test to evaluate the normality of their residuals. The data that did not meet the assumptions of homogeneity and normality were instead analyzed with the Wilcoxon-Mann-Whitney non-parametric test.

### Gut microbiota

For the analysis of the gut microbiota, the intestinal digesta was initially expelled after the opening of the ventral surface of the abdomen, and then the intestinal loops were removed and sectioned into two portions: proximal and distal. By applying gentle pressure to the intestinal loops, the feces were expelled and stored (with microbiota not adhered) in sterile 15 mL Falcon tubes. The intestinal loops were then carefully washed with sterile 0.9% saline solution and stirred at 200 rpm, and the resulting supernatant was removed and separated for collection of the adherent bacteria. The intestinal contents (feces + supernatants) were then placed into sterile Falcon tubes (15 mL) containing sterile 0.9% saline solution. For each diet (CON and SUP), a three-fish digesta pool was established in duplicate. The tubes were placed in a Styrofoam box with dry ice until arrival at the laboratory, where they were stored in a freezer at -80°C.

The commercial PowerSoil^®^ DNA Isolation kit (MO BIO Laboratories, Carlsbad, CA, USA) was used for the extraction of bacterial DNA from the digesta samples, according to the recommendations of the manufacturer. The V3-V4 region of the 16S subunit gene of the bacterial ribosome was amplified with primers from the Illumina platform. The quality of the generated amplicons was verified, and they were then sent to Neoprospecta (Santa Catarina, Brazil) to be sequenced (paired-end library) on the Illumina MiSeq platform with the 250-cycle V2 kit.

Briefly, the samples were normalized to 5 ng uL^-1^, and then polymerase chain reaction (PCR) was performed with Illumina TruSeq adapters (Illumina, San Diego, CA) under the following conditions: 95°C for 5 min, 25 cycles of 95°C for 45 s, 55°C for 30 s, and 72°C for 45 s, and a final extension step at 72°C for 2 min. Subsequently, a second PCR was performed with the index sequences under the following conditions: 95°C for 5 min, 10 cycles of 95°C for 45 s, 66°C for 30 s, and 72°C for 45 s, and a final extension step at 72°C for 2 min. The final product of this PCR was purified with the aid of AMPureXP beads (Beckman Coulter, Brea, CA), and the samples were grouped into sequencing libraries. The libraries were sequenced on a MiSeq system, using the standard Illumina primers provided in kit V2, with 250 cycles at each end.

Bioinformatic analyses were performed using the Mothur software (v.1.36.1) following the methodologies described by [33] and [34], with some modifications. Briefly, contigs were assembled based on the ‘fastq’ files of the ‘read1’ and ‘read2’ produced for each sample. Ambiguities in the sequences were removed, and the sequences were later aligned with the SILVA database. Homopolymers, nucleotide redundancies, nonspecific amplifications, and chimeras were removed with the aid of the VSEARCH algorithm. The sequences were then classified and grouped into OTUs (operational taxonomic units) for the taxonomic comparison of the sequences. To reduce the error caused by non-uniformity in the number of sequences, a subsample was taken from the sample with the lowest number of sequences for the normalization of the data. The Chao, Simpson, Inverse Simpson, and Shannon diversity indices were then calculated, and their mean values were submitted to t-test (significance level of 5%). To compare the structure of the microbial communities between the two treatments, an analysis of molecular variance (AMOVA) was performed. A Venn diagram was generated to demonstrate the intersection of the microbial assemblages among the analyzed pools.

## Results

### Water temperature, mortality, and zootechnical indexes

The mean water temperature was 25.99 ± 3.58°C. There was no statistically significant difference between the mortality of fish fed the SUP and CON diets (p > 0.05). The final weight of tilapia was 732.69 ± 41.17 g in the SUP diet group and 678.90 ± 39.56 g in the CON diet group, which did not significantly differ between diets (p = 0.35). The standard and total lengths were numerically higher in SUP, but without significant difference (p > 0.05). The standard length means (cm) were 25.19 ± 0.43 cm and 24.41 ± 0.41 cm, for SUP and CON (p = 0.20), respectively; and total lengths means were 30.33 ± 0.50 cm and 29.63 ± 0.46 cm (p = 0.32) (SUP and CON, respectively). The condition factor (CF %) was similar between treatments (2.54 ± 0.04 for SUP, and 2.53 ± 0.04 for CON), with no statistical difference (p = 0.87).

### Hematological profile, total serum protein and serum lysozyme

There were no significant differences (p > 0.05) between the fish in the SUP and CON diet groups in their measurements of the VG, Hb, MCV, MCH, and MCHC parameters. Erythrocyte counts (RBC×10^6^ μL^-1^), on the other hand, showed significantly lower values (p = 0.02) in the SUP than in the CON group (Table 3). The differential leukocyte cell counts (×10^3^ μL^-1^) did not show significant differences between diets for total leukocytes (WBC), neutrophils, or monocytes. However, a significant difference (p = 0.04) in lymphocyte counts was observed, with there being lower values in the fish on the SUP diet. No eosinophils and basophils were observed in either of the treatments tested. Thrombocyte counts and total serum protein and serum lysozyme concentrations, also did not differ significantly between the diet groups (p > 0.05) (Table 3).

**Table 3:** Hematological parameters (mean ± standard error), total serum protein, and serum lysozyme concentrations of Nile tilapia fed with a diet containing 1.2% *Schizochytrium* sp. meal (SUP) or a control diet (CON, commercial feed without supplementation) for 105 days.

**Diet**	**RBC** **(x10**^**6**^ **μL**^**-1**^**)**	**Hb** **(g dL**^**-1**^**)**	**Ht** **(%)**	**MCH** **(pg)**	**MCV** **(fL)**	**MCHC** **(g dL**^**-1**^**)**
**CON**	**2.27** **±0.06**	9.67 ±0.11	33.57 ±1.52	43.18 ±1.56	149.11 ±7.49	29.91 ±1.91
**SUP**	**2.06** **±0.05**	9.62 ±0.11	30.64 ±1.29	47.16 ±1.33	149.20 ±5.15	31.94 ±1.04
**p-value**	0.02*	0.73	0.15	0.06	0.99	0.36
	**WBC** **(×10**^**3**^ **μL**^**-1**^**)**	**Lymphocytes** **(×10**^**3**^ **μL**^**-1**^**)**	**Neutrophils (×10**^**3**^ **μL**^**-1**^**)**	**Monocytes (×10**^**3**^ **μL**^**-1**^**)**	**Thrombocytes** **(×10**^**3**^ **μL**^**-1**^**)**	
**CON**	46.91 ±4.13	28.92 ±3.10	13.88 ±2.90	4.10 ±0.77	50.24 ±4.32	
**SUP**	37.42 ±2.06	21.07 ±1.96	11.81 ±2.44	4.54 ±0.91	49.52 ±4.15	
**p-value**	0.05	0.04*	0.59	0.72	0.90	
	**Total serum proteins (g dL**^**-1**^**)**			**Lysozyme (μg mL**^**-1**^**)**		
**CON**	3.58±0.05			16.22±1.17		
**SUP**	3.57±0.07			16.94±2.21		
**p-value**	0.96			0.77		

RBC, red blood cell; Hb, hemoglobin; Ht, hematocrit; MCH, mean corpuscular hemoglobin; MCV, mean corpuscular volume; MCHC, mean corpuscular hemoglobin concentration; WBC, white blood cell.

*p < 0.05: significant at the 5% level.

### Intestinal histology

Regarding intestinal morphology, there were no statistically significant differences in any of the evaluated parameters between the diet groups (Table 4). Numerically, the greatest (but non-significant) differences were found in the number of goblet cells and the presence of supranuclear vacuoles, which both had higher scores in fish in the CON than the SUP diet group (Table 4). S1 Fig demonstrates each of the parameters evaluated by the developed score system, and illustrates the morphological and cellular alterations observed for each characteristic evaluated.

**Table 4:** Histological scores (mean ± standard error) of different aspects of the intestinal morphology of Nile tilapia fed with a diet supplemented with 1.2% *Schizochytrium* sp. meal (SUP) or a control diet (CON).

Diet	MF	LP	SNV	GC	EG
**CON**	2.12 ±0.15	2.06±0.17	2.13±0.09	2.12±0.18	2.06±0.19
**SUP**	2.18±0.13	2.25±0.17	1.87±0.13	1.81±0.16	2.00±0.20
**p-value**	0.80	0.44	0.26	0.21	0.84

MF, mucosal folds; LP, lamina propria; SNV, supranuclear vacuoles; GC, goblet cells; EG, eosinophilic granulocytes.

### Gut microbiota

**Fig 1:**
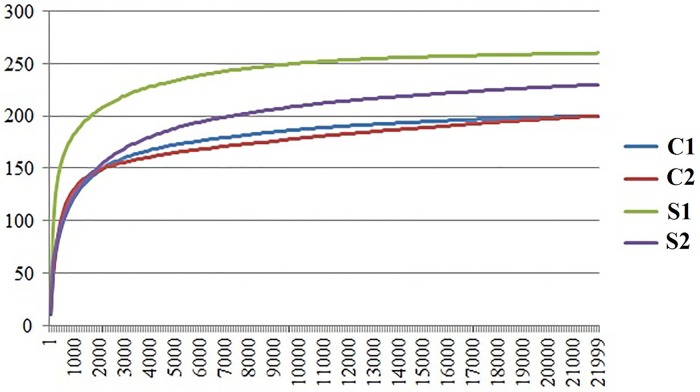
Rarefaction curve of each sample showing the number of reads (x-axis) in relation to the number of OTUs (y-axis). Control group: C1, C2; Supplemented group: S1, S2.

A total of 272,941 contigs were generated in the sequence reads. After quality control, a total of 258,918 contigs were generated, which were aligned in the SILVA database to access information on the OTUs present in the samples. A subsample of 21,999 reads per sample was used for the normalization of the data, which generated the rarefaction curve of the samples shown in Fig 1. The subsample yielded coverage higher than 99.9%, indicating good representativeness of the total microbial population. After the subsampling, a total of 51 OTUs (operational taxonomic units) were obtained at the genus level, with 37 genera found in both treatments.

The mean alpha diversity indices and their standard errors are shown in Table 5. Significantly higher values of the Chao richness index were observed in the SUP diet group than in the CON group (p < 0.05). Although this group also presented higher values of the Inverse Simpson and Shannon indices, these differences were not statistically significant (p > 0.05). The values of the Simpson index also did not differ between diets (p > 0.05).

**Table 5:** Mean ± standard error of the Chao, Simpson, Inverse Simpson, and Shannon diversity indices of the gut microbiota of Nile tilapia fed the control diet (CON) or that supplemented with 1.2% *Schizochytrium* sp meal (SUP).

Diet	Chao	Simpson	Inverse Simpson	Shannon
**CON**	20.75±7.50	0.82±0.06	1.22±0.14	0.45±0.11
**SUP**	26.00±0.00	0.57±0.12	1.82±0.55	0.97±0.12
**p-value**	0.02*	0.21	0.27	0.08

*p < 0.05: significant the 5% level.

**Fig 2:**
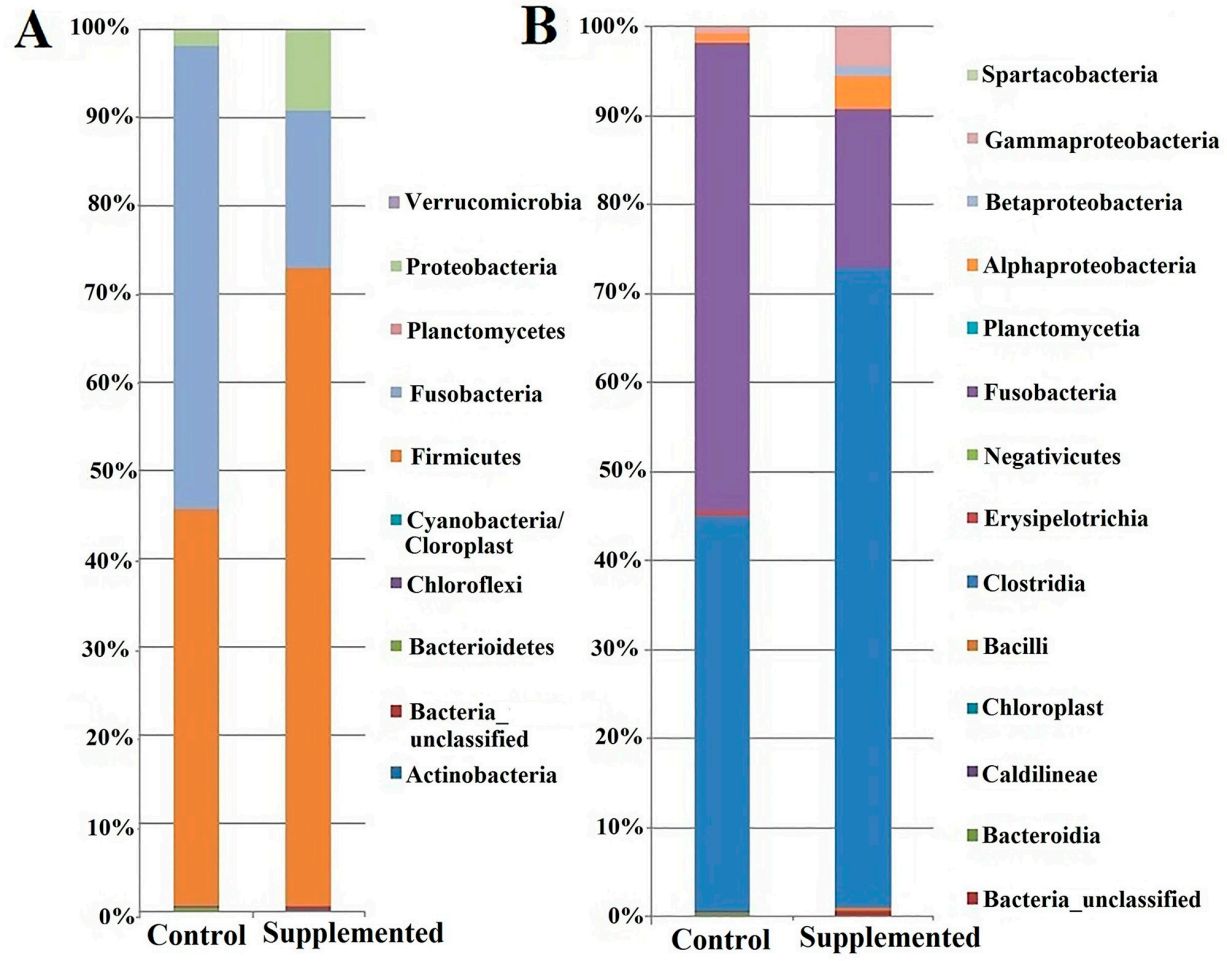
Mean relative abundances of different OTUs of gut bacteria in Nile tilapia fed with a diet containing 1.2% *Schizochytrium* sp. meal (SUP) or a control diet (CON). Results are shown at the levels of bacterial (A) phyla and (B) classes.

The relative abundances of different bacterial OTUs at the phylum and class levels in each treatment are shown in Fig 2. Members of 10 different phyla were found, with the phyla Firmicutes, Fusobacteria, and Proteobacteria being predominant (Fig 2A). In the SUP diet group, there was a predominance of the phylum Firmicutes, whereas in the CON group the relative abundances of Firmicutes and Fusobacteria were balanced. Proteobacteria was the third most abundant phylum for both diets tested.

At the class level (Fig 2B), there was a predominance of the class Clostridia (Firmicutes) in the SUP diet group, and of Fusobacteriia (Fusobacteria) and Clostridia (Firmicutes) in the CON group. The SUP diet group had a higher relative abundance of Alphaproteobacteria, Betaproteobacteria, and Gammaproteobacteria (Proteobacteria) in relation to that in the CON diet group. In turn, the presence of several exclusive classes in the CON group, such as Erysipelotrichia and Negativicutes (Firmicutes), Bacteroidia (Bacteroidetes), and Caldilineae (Chloroflexi), was found (Fig 2B). However, the SUP diet group had only one exclusive class, Bacillus (Firmicutes). Representatives of other classes, such as Planctomycetia (Planctomycetes) and Spartobacteria (Verrucomicrobia), were found only at low relative abundances in both diet groups.

Of the 51 genera identified, the relative abundances of the 15 orders and genera with the highest numbers of sequences are displayed in S1 Table 1. The genus *Romboutsia* (phylum Firmicutes) and *Cetobacterium* (Fusobacteria) were the most abundant in both treatments (128,238 and 117,108 sequences in total, respectively), followed by *Fusobacteriace*_unclassified (3254), *Pseudomonas* (2195), and *Methylocystis* (1731). The *Romboutsia* genus was the one that presented the highest relative abundance in the SUP diet group (71.76%), while in the CON group, the genus *Cetobacterium* was the most abundant one (51.83%).

The analysis of molecular variance (AMOVA) demonstrated that there was greater variation within treatments than between them, and thus found a non-significant structuring between diets (p > 0.05). The Venn diagram found a total of 51 OTUs, with 14 exclusive genera for each treatment and 23 shared genera (S2 Fig).

## Discussion

The mean temperature of the water during the experimental period was compatible with the thermal comfort zone of Nile tilapia [6], and was similar to that in a previous experiment conducted in Rio do Corvo [4]. The zootechnical indexes of tilapia did not differ significantly between treatments (p > 0.05), and the final weight was consistent with the slaughter weight of this species when farmed in net cages.

Recent studies have been carried out to evaluate the effects of diet on the hematological, immunological, and histological parameters or the gut microbiota of Nile tilapia [5,8,35–41], which have contributed to our understanding of the effects of dietary constituents on different physiological aspects of this species. However, no study to date has evaluated these variables in an integrated manner and/or in real farming situations. Despite the absence of significant effects of the diet tested in this study on the final weight and mortality of tilapia, its influence on hematological parameters and the gut microbiota revealed that the addition of 1.2% *Schizochytrium* sp. meal to the diet may influence important aspects of the health of Nile tilapia.

Wide variation in the hematological parameters of Nile tilapia is found in the literature due to a variety of factors, such as alterations in temperature [42], the administration of vaccines [43], stress stimuli [44], and supplementation of diets with probiotics [37,38] and algae [8,18], among others. The values of the hematological parameters found in the present study were similar to those in the studies cited above.

The lower erythrocyte counts found in the SUP diet group may have been related to physiological processes resulting from microalgae ingestion, which may be related to stress minimization, as already shown in studies with other food additives provided for tilapia. In a study by [38], Nile tilapia at high stocking densities showed higher RBC counts in the control group than in the group receiving a probiotic (*Bacillus subtilis*) in their diet. The authors of that study emphasized that the supplied probiotic minimized the erythrocytosis related to the physiological stress caused by the high density, which increased the fish’s oxygen supply. Similarly, [44] observed an increase in the number of RBCs in tilapia submitted to single and consecutive stress stimuli. Additionally, [41]found lower RBC values in tilapia that received phytogenic compounds and organic salts compared to the control group. The RBC count in the present study was slightly higher than that found by [41], who considered normal values for the species. Therefore, microalgae supply would hardly be related to anemia processes, corroborated by Ht (%) values above 20% [29].

It is known that animals held at high densities, such as in net cages, can present elevated stress levels [3], which can lead to an increase in the number of red blood cells in the blood to meet the physiological oxygen demand [38]. Glucocorticoids, such as cortisol, promote adaptive changes in cells and tissues in response to stressors, and erythrocytosis is one of the compensatory stress responses [38]. As observed by [38] by providing *Bacillus subtilis*, it is possible that the inclusion of microalgae in the present work has minimized erythrocytosis related to the physiological stress. However, even though lower RBC values were found in the SUP diet group in this study, significant differences were not found (p > 0.05) in the Hb concentration and hematimetric indices between the diets tested. The greater numerical difference in the hematimetric indices, such as the MCH (p = 0.06), indicated the possibility that the amount of hemoglobin present in each erythrocyte was increased [29] in the SUP group, which would demonstrate a physiological compensation of oxygen supply due to lower RBC values, increasing the ability of red blood cells to carry hemoglobin. Future studies with stress markers such as cortisol, glucose, lactate and serum electrolytes may clarify whether microalgae can reduce tilapia stress in net cages.

The differential analysis of WBC counts is an important indicator of fish health and immunity status that provides information on cell defense conditions [38]. The use of dietary algae such as *Gracilaria verrucosa* has been shown to be able to modulate leukocyte cells and increase the survival rate of tilapia following *Streptococcus agalactiae* challenge [19]. In the present study, no statistically significant difference was observed (p > 0.05) in the counts of any leukocytes between diets, except for lymphocytes. Counts of thrombocytes, cells responsible for the formation of protective barriers that also possess phagocytic activity [29,45], also did not differ significantly between diets (p = 0.90). In a study evaluating the effect of the alga *Sargassum ilicifolium* in the diet of a sturgeon (*Huso huso*), it was found that supplying algae at the concentration of 10% of the diet increased the number of WBCs, with an increase in the numbers of neutrophils and a decrease in the number of lymphocytes [46]. In sea bream (*Sabanejewia aurata*) fed with the alga *Asparagopsis taxiformis* and its extract, significant decreases (p < 0.05) in RBC counts, Hb, Ht, and WBC counts were found [47]. That study’s authors suggested that the inclusion of algae in the diet had negative effects on hematological parameters, which could be related to a dose/response effect.

Although there was an apparent decrease in numbers of WBCs (p = 0.05) suggested by changes in lymphocyte counts, the findings of the present study indicated that the inclusion of the alga in the diet did not drastically influence the immune response of Nile tilapia, considering that the great majority of leukocytes, in addition to the concentrations of total serum protein and lysozyme, did not differ between treatments. [46] observed an increase in serum total proteins and lysozyme activity in sturgeon fed with the alga *Sargassum ilicifolium* at concentrations of 7.5 and 10% in relation to the control group. In contrast, Atlantic salmons fed fourteen weeks with the macroalga *Palmaria palmata* at concentrations of 5, 10 and 15% in the diet showed no significant differences in serum total protein values and lysozyme activity compared to the control group [48], as in the present study. These results demonstrate that the immune responses of algae-fed fish can be variable, depending on the algae species, concentrations used, and even fish eating habits, which can affect the ability to digest and efficiently absorb nutrientes [48].

With regard to lymphocytes, PUFAs have been shown to reduce proinflammatory responses, decreasing the ability of T lymphocytes to proliferate [21]. The probable mechanism of this modulation is based on the increased number of suppressor T lymphocytes that occurs with the administration of DHA, which negatively regulates the other lymphocytes [20,21,49]. The presence of high amounts of n-3 PUFAs in the diet supplemented with the microalga *Schizochytrium*, was possibly the reason for the reduction in lymphocyte concentrations seen in the SUP group. More specific immunological assays, such as tests of the expression of CD4 + lymphocytes, FoxP3, and interleukins, could clarify the lymphocyte modulation of diets rich in PUFAs in fish in more detail.

The microbial community occupying the gastrointestinal tracts of fish plays a crucial role in the development, physiology, and health of these animals, since it stimulates the development of the immune system and promotes competition with pathogenic microorganisms, in addition to also being fundamental in preserving the structure and integrity of the intestinal villi to ensure the adequate acquisition and metabolism of nutrients by the fish [15,17]. Regarding the analysis of the gut microbiota in the present study, the rarefaction curve constructed after subsampling demonstrated uniformity in the number of individuals detected in relation to the number of reads in the samples (Fig 1). The coverage being greater than 99.9% of all samples demonstrated that the sequences obtained were sufficient to represent most of the total microbial diversity, which strengthens the reliability of the results. Although the Chao richness index presented significantly higher values in the SUP diet group (p < 0.05), the treatments did not differ significantly in their values of the Simpson, Inverse Simpson, and Shannon indices, which indicate the alpha diversity of the bacterial community. This result was probably caused by the different abundances of bacterial species between the fish in the CON and SUP diet groups, which did not significantly influence species heterogeneity and equitability.

In a study of rainbow trout (*Oncorhynchus mykiss*) fed with the microalga *Schizochytrium limacinum* for 15 weeks, [13] observed higher numbers of OTUs and Chao index values in the supplemented group than in the control. These authors pointed out that these results may have been due to the adaptation of the gut microbiota to the digestion of the polysaccharides present in the microalga, which was sufficient to cause differences in diversity and richness, but not in the bacterial community structure. In the present study, although there was no difference in the number of OTUs between treatments (37 in each), the highest value of the Chao index found agreed with that found by [13], indicating a greater richness in the bacterial community in the fish that received the diet with the microalga, and the absence of effects on the microbial community structure of the diets tested.

The composition of the microbiota being predominantly represented by the phyla Firmicutes, Fusobacteria, and Proteobacteria corroborates the findings of other studies of the gut microbiota of Nile tilapia [35,50,51], although with different abundances of each phylum found. A slight increase in the relative abundance of Firmicutes was also observed in trout that received a diet containing *Schizochytrium limacinum* [13]. Increased bacterial richness and diversity, in addition to increased abundance of the phylum Firmicutes, has also been related to increased consumption of the proteins and carbohydrates in vegetable flour, which includes lactic acid bacteria (LAB) [52]. These bacteria are abundant in the gut microbiota of some fish species, and are associated with benefits to the intestinal epithelium and improved immune responses [15,53,54]. Even though bacteria of the order Lactobacillales were present only in the supplemented group in the present study, they were only the 15^th^ most abundant bacterial taxon in this group (S1 Table), and were predominantly represented by the Clostridiales. Therefore, the LAB likely did not have a significant influence on the percentage of Firmicutes found in this study.

There is wide variation in the bacterial genera present in the intestinal microbiota among teleosts. However, the genus *Cetobacterium*, which was abundant in both treatments in the present study, is highly representative of the gut microbiota in other fishes, such as in some carp species [16], channel catfish [55], rainbow trout [13], and even species of tilapia [35,51]. This bacterial genus is apparently commensal and encompasses the core microbiota of these species. Its importance is related to its capacity to synthesize vitamin B_12_, antimicrobial metabolites, and volatile fatty acids, mainly acetic acid [56,57]. *Romboutsia* is another widely abundant genus of bacteria that was found mainly in the SUP group in the present study, which is adapted to an environment rich in carbohydrates and exogenous sources of amino acids, and is able to use these before other bacterial taxa by different metabolic pathways [58]. Carbon sources, such as glucose, fructose, maltose, trehalose, xylose, and sorbitol, can be used by different strains of this bacterium [58,59], which can synthesize acetic acid, ethanol, isso-butanoic acid, and isso-valeric acid via glucose fermentation [60].

The presence of saccharides, such as xylose and glucose, in addition to galactose and mannose, in the cell wall of the microalga *Schizochytrium* [61,62] may have contributed to the energetic supply of bacteria of the genus *Romboutsia*, which would have favored the proliferation and increased abundance of these bacteria. However, the absence of *in vivo* studies on the use of these nutrients and the modulation of gut microbiota hampers the ability for precise conclusions to be made about the influence of these compounds on this bacterial genus or other abundant ones. Investigations into the mechanisms of energy utilization by different gut bacteria of fish could more clearly elucidate the mechanisms of the modulation of gut microbiota by exogenous sources of nutrients.

Another genus abundant in both treatments, but mainly in the treated group, *Pseudomonas* has already been classified as one of the dominant genera in fish microbiota [63,64]. This genus is also found in freshwater aquatic environments and is rarely reported as pathogens in these animals, being a potential antagonist of pathogenic microorganisms from bacterial and fungal origin [64]. Less abundant and exclusive bacteria from the CON group such as bacteria from the genus *Turicibacter* and family *Caldilineaceae* have been reported in fish microbiota in natural environments [65,66]. Another bacteria little present, but with greater abundance in the SUP group, *Ralstonia* has been found in greater abundance in sea bass fed on functional diets, and its dominance possibly justified by the presence of essential oils in the diet [67]. The *Escherichia-Shigella* group, although considered pathogenic to other hosts, apparently have commensal characteristics in fish [68]. It should be emphasized that this was the first study that evaluated the gut microbiota of tilapia cultivated in net cages, and thus it can lead to further research the culture environment. For this reason, the composition of the microbial community and the abundance of certain genera found differed from those in other experiments performed in laboratory conditions, although it should be noted that the ‘core’ microbiota of the studied species were apparently maintained. Therefore, the present results may serve as a basis for future research on experimental conditions in the field to help researchers better understand the intestinal microbial ecology of fish in different growing environments.

The intestinal epithelial cells act as a first line of defense against potentially harmful agents, while also ensuring the adequate nutrient utilization, immune defense, and growth of fish [69]. The absence of significant difference (p > 0.05) in the intestinal morphology between the fish fed the CON and SUP diets indicated that the presence of the microalga in the digestive tract and the modulation of the gut microbiota caused by it was not enough to modify the structure and integrity of the intestinal villi, which eliminates the possibility of enteritis processes caused by algae. The score developed may be useful for future histological approaches in studies of teleosts, particularly Nile tilapia.

In recent years, new natural compounds, such as algae and phytogenic extracts, have been extensively explored as viable economic alternatives for use in animal production, including aquaculture. Added to this, there is a growing global demand for research on compounds that pose no risks to the environment and the consumer, but which can have physiological benefits to the cultured animals, such as promoting the better utilization of nutritional components and the maintenance of general health. In conclusion, the results of the present study indicate that the microalga *Schizochytrium* sp. has the capacity to modulate blood cells, including red blood cells and lymphocytes, and also has the potential to manipulate the intestinal microbial community, without having effects on the structure and integrity of the intestinal villi. Further studies with different concentrations of *Schizochytrium* sp., under other farming conditions, and using different increment of transcriptomic analyses could profoundly clarify the modulatory capacity of this microalga on the physiology of Nile tilapia.

## Supporting information

S1 Fig Intestinal morphology of Nile tilapia (*Oreochromis niloticus*). GC, goblet cells; LP; lamina propria; EG, eosinophilic granulocytes; SNV, supranuclear vacuoles; LV, lymphatic vessel. (A) Epithelium and whole villi, with no signs of flattening; fine and complete LP; and GC in small amounts. (B) Increase in CG; LP with slight thickening; diffuse reduction of SNV. (C) GC increased. (D) Shrinkage of villi; LP with increased size; SNV present and aligned. (E) ‘Crumbling’ (disruption) of villi; LV present; absence of SNV. (F) Increased presence of EG in LP; SNV present and aligned. (A, B, C, D, E: Alcian blue staining; F: H&E staining). Scale bar = 50 μm.
(TIF)

S2 Fig Venn diagram showing the intersection between the bacterial diversity in the gut microbiota of the control (CON) and supplemented (SUP) diet groups. (TIF)

S1 Table Relative abundances (at order and genus level) of the 15 most abundant gut bacteria of Nile tilapia fed with a diet containing 1.2% *Schizochytrium* sp. meal (SUP) or a control diet (CON). (DOCX)

## References

[1] FAO. The State of World Fisheries and Aquaculture 2018—Meeting the sustainable development goals [Internet]. Vol. 35. 2018. 176 p. ftp://ftp.fao.org/docrep/fao/011/i0250e/i0250e.pdf

[2] IBGE. Pesquisa da Pecuária Municipal—PPM [Internet]. 2017 [cited 2019 May 1]. https://www.ibge.gov.br/estatisticas-novoportal/economicas/agricultura-e-pecuaria/9107-producao-da-pecuaria-municipal.html?=&t=resultados

[3] Adamante WB, Nuñer APO, Barcellos LJG, Soso AB, Finco JA. Stress in Salminus brasiliensis fingerlings due to different densities and times of transportation. Arq Bras Med Vet e Zootec Med veterinária e Zootec. 2008;60(3):755–61.

[4] Buck EL, Mizubuti IY, Alfieri AA, Otonel RAA, Buck LY, Souza FP, et al Effect of propolis ethanol extract on myostatin gene expression and muscle morphometry of Nile tilapia in net cages. Genet Mol Res. 2017;16(1):1–13. 10.4238/gmr16019404 28362981

[5] Levy-Pereira N, Yasui GS, Cardozo MV, Dias J, Neto, Farias THV, et al Revista Brasileira de Zootecnia Immunostimulation and increase of intestinal lactic acid bacteria with dietary mannan-oligosaccharide in Nile tilapia juveniles. Rev Bras Zootec. 2018;47:e20170006.

[6] Kubitza F. Tilápia: tecnologia e planejamento na produção comercial. 2nd ed Jundiaí: Acqua Supre; 2011 316 p.

[7] Turco PHN, Donadelli A, Scorvo CMDF, JDS, Tarsitano MAA. Análise econômica da produção de Tilápia em tanques-rede de pequeno volume: manejo de ração com diferentes teores de proteína bruta. Informações Econômicas. 2014;44(1):5–11.

[8] Quezada-Rodríguez PR, Fajer-Ávila EJ. The dietary effect of ulvan from Ulva clathrata on hematological-immunological parameters and growth of tilapia (Oreochromis niloticus). J Appl Phycol. 2017;(29):423–31.

[9] Norambuena F, Hermon K, Skrzypczyk V, Emery JA, Sharon Y, Beard A, et al Algae in fish feed: Performances and fatty acid metabolism in juvenile Atlantic Salmon. PLoS One. 2015;10(4):1–17. 10.1371/journal.pone.0124042 PMC4398455 25875839

[10] Lewis TE, Nichols PD, McMeekin TA. The biotechnological potential of thraustochytrids. Mar Biotechnol. 1999;1(6):580–7. 10.1007/pl00011813 10612683

[11] Sarker PK, Kapuscinski AR, Lanois AJ, Livesey ED, Bernhard KP, Coley ML. Towards sustainable aquafeeds: Complete substitution of fish oil with marine microalga Schizochytrium sp. improves growth and fatty acid deposition in juvenile Nile tilapia (Oreochromis niloticus). PLoS One. 2016;11(6):1–17. 10.1371/journal.pone.0156684 PMC4892564 27258552

[12] Li MH, Robinson EH, Tucker CS, Manning BB, Khoo L. Effects of dried algae Schizochytrium sp., a rich source of docosahexaenoic acid, on growth, fatty acid composition, and sensory quality of channel catfish Ictalurus punctatus. Aquaculture [Internet]. 2009;292(3–4):232–6. Available from: 10.1016/j.aquaculture.2009.04.033

[13] Lyons PP, Turnbull JF, Dawson KA, Crumlish M. Effects of low-level dietary microalgae supplementation on the distal intestinal microbiome of farmed rainbow trout Oncorhynchus mykiss (Walbaum). Aquac Res. 2017;48(5):2438–52.

[14] dos Santos SKA, Guilherme de Souza Moura MMP, Aline Danielle Souza Prates ALF, Azevedo RC. Microalga Schizochytrium sp. em Rações para Tilápia do Nilo. Cad Ciências Agrárias. 2015;7(1):75–9.

[15] Dimitroglou A, Merrifield DL, Moate R, Davies SJ, Spring P, Sweetman J, et al Dietary mannan oligosaccharide supplementation modulates intestinal microbial ecology and improves gut morphology of rainbow trout, Oncorhynchus mykiss (Walbaum). J Anim Sci. 2009;87(10):3226–34. 10.2527/jas.2008-1428 19617514

[16] Eichmiller JJ, Hamilton MJ, Staley C, Sadowsky JM, Sorensen PW. Environment shapes the fecal microbiome of invasive carp species. Microbiome. 2016;44(4):1–13. 10.1186/s40168-016-0190-1 PMC4981970 27514729

[17] Tarnecki AM, Burgos FA, Ray CL, Arias CR. Fish intestinal microbiome: diversity and symbiosis unravelled by metagenomics. J Appl Microbiol. 2017;123(1):2–17. 10.1111/jam.13415 28176435

[18] Garcia F, Schalch SHC, Onaka EM, Fonseca FS, Batista MP. Hematologia de tilápia-do-nilo alimentada com suplemento à base de algas frente a desafios de estresse agudo e crônico. Arq Bras Med Vet e Zootec. 2012;64(1):198–204.

[19] Kumala FB, Wahjuningrum D, Setiawati M. Effects of dietary algae, fungi and herb on the growth and innate immunity of Nile tilapia Oreochromis niloticus challenged with Streptococcus agalactiae. AACL Bioflux. 2018;11(4):1368–77.

[20] Kim CH. FOXP3 and its role in the immune system In: Advances in Experimental Medicine and Biology. New York, NY: Springer; 2009 p. 17–29. 10.1007/978-1-4419-1599-3_2 20429413

[21] Chapkin RS, Kim W, Luptona JR., McMurraya DN. Dietary docosahexaenoic and eicosapentaenoic acid: Emerging mediators of inflammation. Prostaglandins Leukot Essent Fat Acids. 2009;81(2–3):187–91. 10.1016/j.plefa.2009.05.010 PMC2755221 19502020

[22] Silva DJ, de Queiroz AC. Analise de Alimentos: Métodos Químicos e Biológicos. 3rd ed Viçosa: UFV; 2002 235 p.

[23] Blight EG, Dyer WJ. A rapid method of total lipid extraction and purification. Can J Biochem Physiol [Internet]. 1959;37(8):911–7. Available from: http://scholar.google.com/scholar?hl=en&btnG=Search&q=intitle:Canadian+Journal+of+Biochemistry+and+Physiology#0 13671378 10.1139/o59-099

[24] Joseph JD., Ackman RG. Capillary column gas chromatography method for analysis of encapsulated fish oil and fish oil ethyl esters: collaborative study. J Assoc Off Anal Chem Int. 1992;75:488–506.

[25] Visentainer JV. Aspectos analíticos da resposta do detector de ionizaçã o em chama para ésteres de ácidos graxos em biodiesel e alimentos. Quim Nova. 2012;35(2):274–9.

[26] De Oliveira SN, Ribeiro RP, de Oliveira CAL, Lopera-Barrero NM, da SO Zardin AM, de Souza FP, et al Multivariate analysis using morphometric and ultrasound information for selection of tilapia (Oreochromis niloticus) breeders. R Bras Zootec. 2019;48:e20170179.

[27] Dawood MAO, Koshio S, El-sabagh M, Billah M, Zaineldin AI, Mamdouh M, et al Changes in the growth, humoral and mucosal immune responses following β-glucan and vitamin C administration in red sea bream, Pagrus major. Aquaculture [Internet]. 2017;470:214–22. Available from: 10.1016/j.aquaculture.2016.12.036

[28] Blaxhall PC, Daisley KW. Routine haematological methods for use with fish blood. J Fish Biol. 1973;5(6):771–81.

[29] Ranzani-Paiva MJT, de Pádua SB, Tavares-Dias M, Egami MI. Métodos para análise hematológica em peixes. Maringá: Eduem; 2013 140 p.

[30] Ellis AE. Lysozyme Assays In: Stolen JS, Fletcher TC, Anderson DP, Roberson BS, Van Muiswinkel WB, editors. Techniques in Fish Immunology. SOS Publications: SOS Publications; 1990 p. 101–3.

[31] Uran PA, Schrama JW, Rombout JHWM, Obach A, Jensen L, Koppe W, et al Soybean meal-induced enteritis in Atlantic salmon(Salmo salar L.) at different temperatures. Aquac Nutr. 2008;14(4):324–30.

[32] R Core Team. R: A language and environment for statistical computing [Internet]. R Foundation for Statistical Computing. 2017 [cited 2018 Nov 11]. https://www.r-project.org/

[33] Schloss PD, Westcott SL, Ryabin T, Hall JR, Hartmann M, Hollister EB, et al Introducing mothur : Open-source, Platform-Independent, Community-Supported Software for Describing and Comparing Microbial Communities. Appl Environ Microbiol. 2009;75(23):7537–41. 10.1128/AEM.01541-09 19801464 PMC2786419

[34] Kozich JJ, Westcott SL, Baxter NT, Highlander SK, Schloss PD. Development of a Dual-Index Sequencing Strategy and Curation Pipeline for Analyzing Amplicon Sequence Data on the MiSeq. Appl Environ Microbiol. 2013;79(17):5112–20. 10.1128/AEM.01043-13 23793624 PMC3753973

[35] Fan L, Chen J, Meng S, Song C, Qiu L, Hu G. Characterization of microbial communities in intensive GIFT tilapia (Oreochromis niloticus) pond systems during the peak period of breeding. Aquac Res. 2017;2007(48):459–72.

[36] Standen BT, Peggs DL, Rawling MD, Foey A, Davies SJ, Santos GA, et al Dietary administration of a commercial mixed-species probiotic improves growth performance and modulates the intestinal immunity of tilapia, Oreochromis niloticus. Fish Shellfish Immunol. 2016;49:427–35. 10.1016/j.fsi.2015.11.037 26672904

[37] Standen BT, Rawling MD, Davies SJ, Castex M, Foey A, Gioacchini G, et al Probiotic Pediococcus acidilactici modulates both localised intestinal- and peripheral-immunity in tilapia (Oreochromis niloticus). Fish Shellfish Immunol. 2013;35:1097–104. 10.1016/j.fsi.2013.07.018 23871840

[38] Telli GS, Ranzani-Paiva MJT, Dias D de C, Sussel FR, Ishikawa CM, Tachibana L. Dietary administration of Bacillus subtilis on hematology and non-specific immunity of Nile tilapia Oreochromis niloticus raised at different stocking densities. Fish Shellfish Immunol. 2014;39(2):305–11. 10.1016/j.fsi.2014.05.025 24878743

[39] Valladão GMR, Gallani SU, Pala G, Jesus RB, Kotzent S, Costa JC, et al Practical diets with essential oils of plants activate the complement system and alter the intestinal morphology of Nile tilapia. Aquac Res. 2017;48(11):5640–9.

[40] Zahran E, Risha E, AbdelHamid F, Mahgoub HA, Ibrahim T. Effects of dietary Astragalus polysaccharides (APS) on growth performance, immunological parameters, digestive enzymes, and intestinal morphology of Nile tilapia (Oreochromis niloticus). Fish Shellfish Immunol [Internet]. 2014;38(1):149–57. Available from: 10.1016/j.fsi.2014.03.002 24657260

[41] Suphoronski SA, Chideroli RT, Facimoto CT, Mainardi RM, Souza FP, Lopera-Barrero NM, et al Effects of a phytogenic, alone and associated with potassium diformate, on tilapia growth, immunity, gut microbiome and resistance against francisellosis. Sci Rep. 2019;9(6045):1–14. 30988331 10.1038/s41598-019-42480-8 PMC6465292

[42] Araujo DDM, Pezzato AC, Barros MM. Hematology of Nile tilapia fed diets with vegetable oils and stimulated by cold. Pesqui Agropecu Bras. 2011;46(3):294–302.

[43] Silva BC, Martins ML, Jatobá A, Buglione CC, Vieira FN, Pereira GV, et al Hematological and immunological responses of Nile tilapia after polyvalent vaccine administration by different routes. Pesqui Veterinária Bras. 2009;29(11):874–80.

[44] Martins ML, Pilarsky F, Onaka EM, Nomura DT, F J Jr, Ribeiro K, et al Hematologia e resposta inflamatória aguda em Oreochromis niloticus (Osteichthyes: Cichlidae) submetida aos estímulos único e consecutivo de estresse de captura. Bol do Inst Pesca. 30(1):71–80.

[45] Tavares-dias BM, Ono EA, Pilarski F, Moraes FR. Can thrombocytes participate in the removal of cellular debris in the blood circulation of teleost fish? A cytochemical study and ultrastructural analysis. J Appl Ichthyol. 2007;23:709–12.

[46] Yeganeh S, Adel M. Effects of dietary algae (Sargassum ilicifolium) as immunomodulator and growth promoter of juvenile great sturgeon (Huso huso Linnaeus, 1758). J Appl Phycol. 2018;1–10.

[47] Marino F, Di Caro G, Gugliandolo C, Spanò A, Faggio C, Genovese G, et al Preliminary Study on the In vitro and In vivo Effects of Asparagopsis taxiformis Bioactive Phycoderivates on Teleosts. Front Physiol. 2016;7:1–11. 27826246 10.3389/fphys.2016.00459 PMC5078491

[48] Wan AHL, Soler-vila A, Keeffe DO, Casburn P, Fitzgerald R, Johnson MP. The inclusion of Palmaria palmata macroalgae in Atlantic salmon (Salmo salar) diets: effects on growth, haematology, immunity and liver function. J Appl Phycol [Internet]. 2016;28:3091–100. Available from: 10.1007/s10811-016-0821-8

[49] Han SC, Koo DH, Kang NJ, Yoon WJ, Kang GJ, Kang HK, et al Docosahexaenoic acid alleviates atopic dermatitis by generating tregs and IL-10/TGF-β-modified macrophages via a TGF-β-dependent mechanism. J Invest Dermatol [Internet]. 2015;135(6):1556–64. Available from: 10.1038/jid.2014.488 25405323

[50] Kohl KD, Amaya J, Passement CA, Dearing MD, Mccue MD. Unique and shared responses of the gut microbiota to prolonged fasting: a comparative study across five classes of vertebrate hosts. Microbiol Ecol. 2014;90:883–94. 10.1111/1574-6941.12442 25319042

[51] Zhang M, Sun Y, Liu Y, Qiao F, Chen L, Liu W, et al Response of gut microbiota to salinity change in two euryhaline aquatic animals with reverse salinity preference. Aquaculture [Internet]. 2016;454:72–80. Available from: 10.1016/j.aquaculture.2015.12.014

[52] Desai AR, Links MG, Collins SA, Mansfield GS, Drew MD, Van Kessel AG, et al Effects of plant-based diets on the distal gut microbiome of rainbow trout (Oncorhynchus mykiss). Aquaculture. 2012;350–353:134–42.

[53] Panigrahi A, Kiron V, Kobayashi T, Puangkaew J, Satoh S, Sugita H. Immune responses in rainbow trout Oncorhynchus mykiss induced by a potential probiotic bacteria Lactobacillus rhamnosus JCM 1136. Vet Immunol Immunopathol. 2004;102(4):379–88. 10.1016/j.vetimm.2004.08.006 15541791

[54] Reveco FE, Øverland M, Romarheim OH, Mydland LT. Intestinal bacterial community structure differs between healthy and inflamed intestines in Atlantic salmon (Salmo salar L.). Aquaculture [Internet]. 2014;420–421:262–9. Available from: 10.1016/j.aquaculture.2013.11.007

[55] Bledsoe JW, Peterson BC, Swanson KS, Small BC. Ontogenetic characterization of the intestinal microbiota of channel catfish through 16S rRNA gene sequencing reveals insights on temporal shifts and the influence of environmental microbes. PLoS One. 2016;11(11):1–22. 10.1371/journal.pone.0166379 PMC5113000 27846300

[56] Finegold SM, Vaisanen ML, Molitoris DR, Tomzynski TJ, Song Y, Liu C, et al Cetobacterium somerae sp. nov. from human feces and emended description of the genus Cetobacterium. Syst Appl Microbiol. 2003;26(2):177–81. 10.1078/072320203322346010 12866843

[57] Tsuchiya C, Sakata T, Sugita H. Novel ecological niche of Cetobacterium somerae, an anaerobic bacterium in the intestinal tracts of freshwater fish. Lett Appl Microbiol. 2008;46(1):43–8. 10.1111/j.1472-765X.2007.02258.x 17944860

[58] Gerritsen J, Hornung B, Renckens B, Van Hijum SAFT, Martins VAP, Rijkers GT, et al Genomic and functional analysis of Romboutsia ilealis CRIB T reveals adaptation to the small intestine. PeerJ. 2017;5:1–28. 10.7717/peerj.3698 PMC5598433 28924494

[59] Gerritsen J, Fuentes S, Grievink W, van Niftrik L, Tindall BJ, Timmerman HM, et al Characterization of Romboutsia ilealis gen. nov., sp. nov., isolated from the gastro-intestinal tract of a rat, and proposal for the reclassification of five closely related members of the genus Clostridium into the genera Romboutsia gen. nov., Intestinib. Int J Syst Evol Microbiol. 2014;64(PART 5):1600–16. 24480908 10.1099/ijs.0.059543-0

[60] Wang Y, Song J, Zhai Y, Zhang C, Gerritsen J, Wang H, et al Romboutsia sedimentorum sp. nov., isolated from an alkaline-saline lake sediment and emended description of the genus Romboutsia. Int J Syst Evol Microbiol. 2015;65(2015):1193–8. 25609678 10.1099/ijs.0.000079

[61] Chamberlain AHL, Moss ST. The thraustochytrids: a protist group with mixed affinities. BioSystems. 1988;21(3–4):341–9. 10.1016/0303-2647(88)90031-7 3395686

[62] Darley WM, Porter D, Fuller MS. Cell wall composition and synthesis via Golgi-directed scale formation in the marine eucaryote, Schizochytrium aggregatum, with a note on Thraustochytrium sp. Arch Mikrobiol. 1973;90(2):89–106. 10.1007/bf00414512 4350550

[63] Etyemez M, Balcázar JL. Bacterial community structure in the intestinal ecosystem of rainbow trout (Oncorhynchus mykiss) as revealed by pyrosequencing-based analysis of 16S rRNA genes. Res Vet Sci [Internet]. 2015;100:8–11. Available from: 10.1016/j.rvsc.2015.03.026 25843896

[64] Gram L, Melchiorsen J, Spanggaard B, Huber I, Al GET, Icrobiol APPLENM. AH2, a Possible Probiotic Treatment of Fish. Appl Env Microbiol. 1999;65(3):969–73. 10049849 10.1128/aem.65.3.969-973.1999 PMC91130

[65] Baldo L, Riera JL, Tooming-Klunderud A, Albà MM, Salzburger W. Gut Microbiota Dynamics during Dietary Shift in Eastern African Cichlid Fishes. PLoS One. 2015;1–23. 10.1371/journal.pone.0127462 PMC4433246 25978452

[66] Tan CK, Natrah I, Suyub IB, Edward MJ, Kaman N, Samsudin AA. Comparative study of gut microbiota in wild and captive Malaysian Mahseer (Tor tambroides). Microbiologyopen. 2019;8(e734):1–12. 10.1002/mbo3.734 PMC6528585 30353678

[67] Carda‐Diéguez M, Mira A, Fouz B. Pyrosequencing survey of intestinal microbiota diversity in cultured sea bass (Dicentrarchus labrax) fed functional diets. FEMS Microbiol Ecol. 2014;87(2014):451–459. 24730648 10.1111/1574-6941.12236

[68] Zheng X, Yang R, Hu J, Lin S, Gu Z, Ma Z. The gut microbiota community and antioxidant enzymes activity of barramundi reared at seawater and freshwater. Fish Shellfish Immunol [Internet]. 2019;89:127–31. Available from: 10.1016/j.fsi.2019.03.054 30930278

[69] Bravo-Tello K, Ehrenfeld N, Solı CJ, Hedrera M, Pizarro-guajardo M, Paredes-sabja D. Effect of microalgae on intestinal inflammation triggered by soybean meal and bacterial infection in zebrafish. PLoS One. 2017;12(11):1–13. 10.1371/journal.pone.0187696 PMC5678869 29117213

