# Supplementary figures and images for: Effects of dietary supplementation with a microalga (Schizochytrium sp.) on the hemato-immunological, and intestinal histological parameters and gut microbiota of Nile tilapia in net cages
Source: PLoS One. 2020 Jan 2;15(1):e0226977. doi: 10.1371/journal.pone.0226977 (PMC6940142; doi:10.1371/journal.pone.0226977)

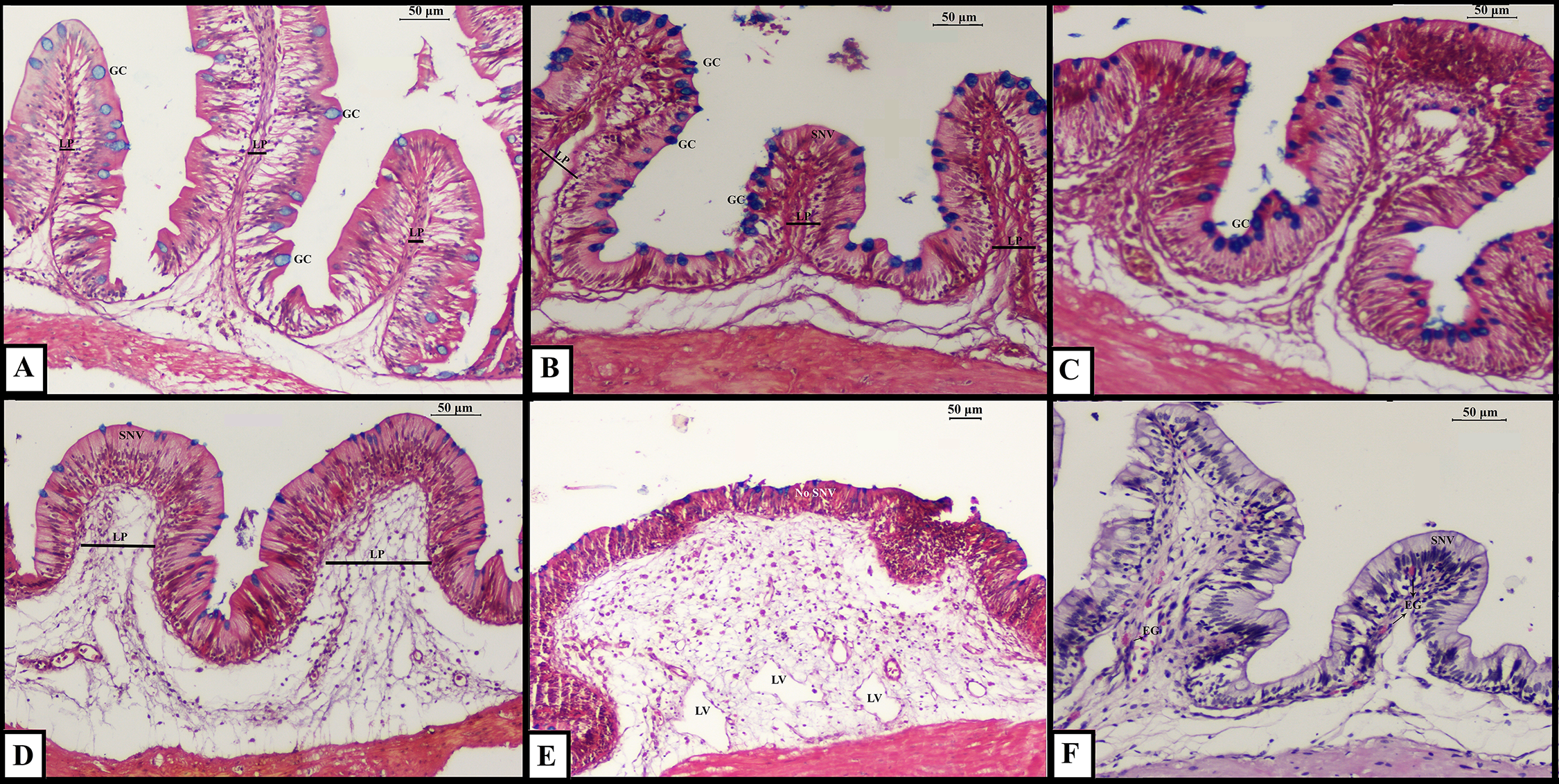

Supplement: S1 Fig — GC, goblet cells; LP; lamina propria; EG, eosinophilic granulocytes; SNV, supranuclear vacuoles; LV, lymphatic vessel. (A) Epithelium and whole villi, with no signs of flattening; fine and complete LP; and GC in small amounts. (B) Increase in CG; LP with slight thickening; diffuse reduction of SNV. (C) GC increased. (D) Shrinkage of villi; LP with increased size; SNV present and aligned. (E) ‘Crumbling’ (disruption) of villi; LV present; absence of SNV. (F) Increased presence of EG in LP; SNV present and aligned. (A, B, C, D, E: Alcian blue staining; F: H&E staining). Scale bar = 50 μm. (TIF) [file pone.0226977.s001.tif]

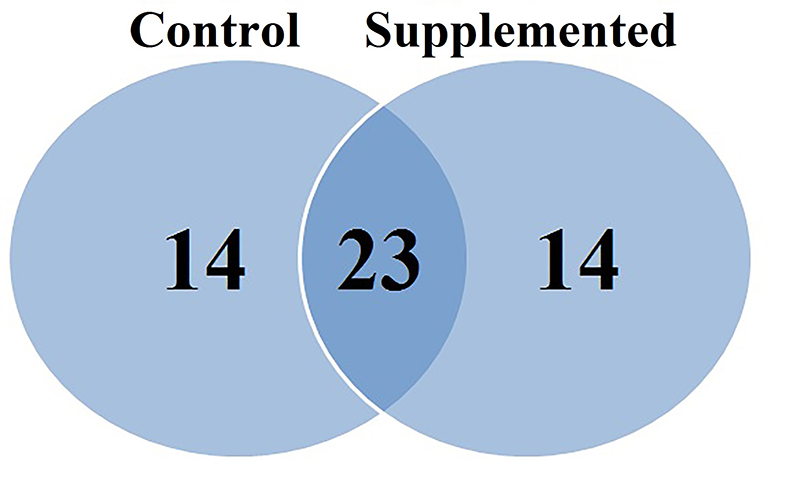

Supplement: S2 Fig — (TIF) [file pone.0226977.s002.tif]
