# Supplementary material for: Effects of dietary supplementation with a microalga (Schizochytrium sp.) on the hemato-immunological, and intestinal histological parameters and gut microbiota of Nile tilapia in net cages
Source: PLoS One. 2020 Jan 2;15(1):e0226977. doi: 10.1371/journal.pone.0226977 (PMC6940142; doi:10.1371/journal.pone.0226977)
Supplement: S1 Table — (DOCX) [file pone.0226977.s003.docx]

**S1 Table. Relative abundances (at order and genus level) of the 15 most abundant gut bacteria of Nile tilapia fed with a diet containing 1.2% *Schizochytrium* sp. meal (SUP) or a control diet (CON).**

| Order | | | Genus | | |  |
| --- | --- | --- | --- | --- | --- | --- |
|  | CON  (%) | SUP(%) |  | CON (%) | SUP (%) | Total of reads |
| Clostridiales | 44.35 | 71.87 | Romboutsia | 44.28 | 71.76 | 128238 |
| Fusobacteriales | 52.6 | 17.86 | Cetobacterium | 51.83 | 14.1 | 117108 |
| Rhizobiales | 0.92 | 3.64 | Fusobacteriaceae  _unclassified | 0.69 | 3.76 | 3254 |
| Pseudomonadales | 0.25 | 3.54 | Pseudomonas | 0.25 | 3.51 | 2195 |
| Erysipelotrichales | 0.55 | 0 | Methylocystis | 0.3 | 2.31 | 1731 |
| Enterobacteriales | 0.27 | 0.13 | Turicibacter | 0.55 | 0 | 1170 |
| Caldilineales | 0.28 | 0 | Rhizobiales  _unclassified | 0.24 | 0.59 | 789 |
| Burkholderiales | 0.04 | 1.05 | Caldilineaceae  _unclassified | 0.28 | 0 | 599 |
| Bacteroidales | 0.22 | 0 | Escherichia/  Shigella | 0.23 | 0.09 | 541 |
| Actinomycetales | 0.17 | 0.17 | Prevotella | 0.22 | 0 | 465 |
| Gamma  proteobacteria | 0.11 | 0.36 | Gamma  proteobacteria  _unclassified | 0.11 | 0.36 | 396 |
| _unclassified |  |  |  |  |  |  |
| Bacteria_  unclassified | 0.04 | 0.46 | Hyphomicrobium | 0.15 | 0 | 320 |
| Legionellales | 0.08 | 0.28 | Hypho  microbiaceae  _unclassified | 0.1 | 0.19 | 311 |
| Lactobacillales | 0 | 0.33 | Bacteria_  unclassified | 0.04 | 0.46 | 304 |
| Rhodospirillales | 0.06 | 0.04 | Ralstonia | 0.04 | 0.48 | 304 |
